# Can nonclinicians classify medication discrepancies as accurately as clinical pharmacists? A validation study

**DOI:** 10.1002/hsr2.824

**Published:** 2022-09-24

**Authors:** Julianne E. Brady, Steven R. Simon, Kate Yeksigian, Alan J. Zillich, Jonathan Moyer, Amy M. Linsky

**Affiliations:** ^1^ Center for Healthcare Organization and Implementation Research (CHOIR) VA Boston Healthcare System Boston Massachusetts USA; ^2^ Center for the Study of Healthcare Innovation, Implementation and Policy VA Greater Los Angeles Healthcare System Los Angeles California USA; ^3^ Department of Medicine, David Geffen School of Medicine University of California Los Angeles Los Angeles California USA; ^4^ Department of Pharmacy Practice, College of Pharmacy Purdue University West Lafayette Indiana USA; ^5^ Office of Disease Prevention National Institutes of Health Bethesda Maryland USA; ^6^ General Internal Medicine VA Boston Healthcare System Boston Massachusetts USA; ^7^ General Internal Medicine Boston University School of Medicine Boston Massachusetts USA

**Keywords:** classification system, medication discrepancy, medication reconciliation, patient safety, quality improvement

## 1 INTRODUCTION

Medication discrepancies, defined as unintended differences between medication lists,^1^ occur in up to 60% of patients' electronic health records (EHRs).^2^, ^3^, ^4^ They are associated with adverse drug events and increased healthcare utilization^5^, ^6^, ^7^, ^8^; thus, the Joint Commission recommends medication reconciliation to resolve discrepancies between patient‐reported medications and those documented within the record.^9^

Medication reconciliation tools to identify and correct discrepancies can provide patient safety and cost‐related benefits.^7^ Discrepancy types within classification systems differ, but common types include Commission (i.e., medication present in EHR but patient not taking), Omission (i.e., medication absent from EHR), and Drug‐dose (i.e., dose missing or incorrect).^10^

The Medication Discrepancy Taxonomy (MedTax) is a universal classification system developed to advance research and evaluations to reduce discrepancies.^1^ MedTax was designed and validated for use by pharmacists only. Exclusively relying on pharmacists' or clinicians' expertise to classify medication discrepancies may be limited by costs and time availability.

Using nonclinicians in medication reconciliation processes could provide many benefits, especially in research and quality improvement (QI) initiatives. However, little is known about the role of nonclinical personnel in the *classification* subprocess. We determined if research assistants (RAs) without formal clinical education can classify identified medication discrepancies with accuracy comparable to pharmacists.

## 2 METHODS

### 2.1 Dataset

A dataset containing 1024 discrepancies (e.g., omissions, commissions) was derived from medication lists of 179 patients (5.7 discrepancies/patient) collected during care transitions as part of a larger trial from December 2019 to October 2020.^11^ All study procedures were approved by the VA Boston Healthcare System Institutional Review Board.

### 2.2 Clinical and educational training of coders

Three coders participated in the classification process. The pharmacist had a Doctor of Pharmacy degree and was completing a postgraduate pharmacy residency. There were two RAs; RA‐1 had a Master of Arts in Psychology, and RA‐2 had a Master of Public Health.

### 2.3 MedTax training procedures

We used a modified version of the validated MedTax^1^ to classify potential medication discrepancies. To modify MedTax, two physicians (Steven R. Simon and Amy M. Linsky) used clinical and content expertise to select medication discrepancy types most applicable to the healthcare setting of the study (see Text Box 1).

Text Box 1 **Modified medication discrepancy taxonomy**

**Table:** 

Type of medication discrepancy	Description
1. Drug omission	Patient reports taking a medication that is omitted from the medical record.
2. Drug commission (or addition)	The medical record includes a medication that the patient reports not taking.
3. Drug duplication	Medication is recorded multiple times in the medical record.
4. The Discrepancy in the strength and/or frequency and/or number of units of dosage form and/or total daily dose	The medical record lists the strength and/or frequency and/or number of units of dosage form and/or total daily dose different from what the patient reports taking.
5. Computer system expiration	Medication listed as “Expired” due to original prescription more than 1‐year‐old, but patient reports still taking.
6. Discrepancy in the dosage, form, and/or route of administration	The medical record lists the dosage, form, and/or route of administration of the medication different from what the patient reports taking.
7. Other	All discrepancies that do not qualify as Types 1–6
8. No discrepancy

A 2‐h training session on the application of the modified MedTax was conducted using 79 medication discrepancies. Participants included three coders: pharmacist (Megan Nowak) and RAs (Kate Yeksigian and Julianne E. Brady) – and two physicians (Steven R. Simon and Amy M. Linsky). During Round 1, all five participants reviewed 25 discrepancies sequentially and collaboratively discussed each classification. After Round 1, the pharmacists, RAs, and physicians independently classified the remaining 54 discrepancies in five training rounds (Rounds 2–6; 13.5 discrepancies/round; range 7–14).

Classification between the three coders and the two physicians (i.e., the “gold standard”) was assessed. Discordant classifications were discussed as a group, with the physicians providing additional guidance. The training aimed to achieve acceptable a priori agreement between the RAs and pharmacist (>75%).^12^

### 2.4 Independent coding session

After the training, the pharmacist and each RA independently coded the remaining 945 discrepancies. The pharmacist reviewed all discrepancies (*n* = 945), and the RAs divided the discrepancies for review (RA‐1: *n* = 516; RA‐2: *n* = 429).

### 2.5 Analysis

#### 2.5.1 Interrater reliability of the training session

To determine the agreement between the pharmacist and two RAs, Krippendorff's *α* was calculated and interpreted using the following benchmarks of agreement: 0–0.667: poor; 0.668–0.799: possibly acceptable; and 0.800–1: acceptable.^13^ To assess individual coder accuracy, Cohen's *κ* was calculated to compare each of the pharmacist and RA coders to the gold standard and interpreted using the following benchmarks of agreement: 0–0.20: poor; 0.21–0.40: fair; 0.41–0.60: moderate; 0.61–0.80: substantial; and 0.81–1.00: almost perfect.^14^ Percent agreement was also calculated.

#### 2.5.2 Interrater reliability of independent coding session

In the primary analysis, we calculated Krippendorff's *α* to measure interrater reliability (IRR) between the three coders. In secondary analyses, we calculated Cohen's *κ* and percent agreement to assess the IRR of each RA to the pharmacist. In supplemental analyses, all IRR statistics for each discrepancy type were calculated. All analyses were conducted using R 4.0^15^ and Stata 16.^16^

## 3 RESULTS

### 3.1 Training session IRR

The RAs and pharmacist showed moderate agreement with each other during the training rounds (*α* = 0.73; 95% confidence interval [CI]: 0.59, 0.83). Across all three coders, the overall percent agreement was 69.8%, just below the a priori goal of 75%.

### 3.2 Independent coding IRR

In the primary analysis, RAs classified discrepancies using the modified MedTax with acceptable accuracy compared to the pharmacist (945 discrepancies, *α* = 0.81, 95% CI: 0.78–0.84). In secondary analyses, both RAs individually coded with acceptable performance compared to the pharmacist, with almost perfect concordance between the pharmacist and both RA‐1 (516 discrepancies; *ƙ* = 0.82, 95% CI: 0.79–0.86; 86.6% agreement) and RA‐2 (429 discrepancies; *ƙ* = 0.80, 95% CI: 0.75–0.84; 84.6% agreement).

In supplemental analyses, the pharmacist and RAs classified five of eight (63%) MedTax medication discrepancy types with acceptable concordance: Omission (*α* = 0.86), Commission (*α* = 0.83), Duplication (*α* = 0.92), Strength (*α* = 0.85), and Computer System Expiration (*α* = 0.92). The pharmacist and RAs had possibly acceptable concordance classifying Drug Dose/Form/Route (*α* = 0.67) and poor concordance classifying No Discrepancy (*α* = 0.65) and Other discrepancies (*α* = 0.31).

A post hoc review of the discordance classifying Other discrepancies found these were all classified as Other by the pharmacist and omission by the RAs. In post hoc blinded physician adjudication of all discordant classifications, the physician agreed with RA classifications for 100% (12/12) of the Other disagreements and 74% (71/96) of the No Discrepancy disagreements.

## 4 DISCUSSION

RAs without formal clinical education can successfully classify medication discrepancies with accuracy comparable to a clinical pharmacist. These findings that RAs can be trained to reliably use a modified medication discrepancy classification taxonomy (MedTax) support engagement of nonclinical individuals to classify discrepancies within research and QI settings (i.e., nonclinical settings) where frequency and types of medication discrepancies are often used to assess the effectiveness of medication reconciliation interventions.^17^ While direct contribution by clinical pharmacists in such efforts is beneficial, competing clinical responsibilities^18^ may preclude their involvement and require identification of nonclinical individuals (e.g., RAs) to perform specific aspects of medication reconciliation.

Employing clinical pharmacists in research and QI projects is difficult due to direct and opportunity costs^19^, ^20^ Cost and time savings, combined with our finding that RA classification is reliable and valid, would yield even greater net benefit when combined with the clinical value of high‐quality medication reconciliation.^21^, ^22^

The RAs classified five of eight discrepancy types (Omission, Commission, Duplication, Strength, Computer System Expiration) with near perfect concordance. Classifying changes in Dosage Form or Route of Administration may be more complex and require additional training. However, post hoc analyses supported an RA's ability to accurately classify these two discrepancy types, despite lower concordance with the pharmacist.

Findings should be interpreted in the context of the following limitations. There were only a small number of individuals; findings should be replicated including coders with more diverse clinical and nonclinical backgrounds. The pharmacist was completing a pharmacy residency; one with more professional experience may yield different findings. This study evaluated the ability to train RAs on one classification system. Nevertheless, the modified taxonomy was based upon the validated MedTax and included classifications for the most common medication discrepancies.

## 5 CONCLUSION

Training RAs to classify medication discrepancies reliably has many benefits in both research and QI settings. Greater availability and lower overall cost of individuals without formal clinical education optimizes the use of limited time and financial resources while maintaining confidence that nonclinical personnel can be trained to classify previously identified medication discrepancies.

## AUTHOR CONTRIBUTIONS

**Julianne E. Brady**: Data curation and writing – original draft preparation. **Steven R. Simon**: Funding acquisition, conceptualization, methodology, and writing – reviewing and editing. **Kate Yeksigian**: Data curation, and writing – reviewing and editing. **Alan J. Zillich**: Conceptualization, methodology, and writing – reviewing and editing. **Jonathan Moyer**: Formal analysis, and writing – reviewing and editing. **Amy M. Linsky**: Conceptualization, methodology, and writing – original draft preparation. All authors have read and approved the final version of the manuscript.

## CONFLICT OF INTEREST

The authors declare no conflict of interest.

## TRANSPARENCY STATEMENT

The lead author Julianne E. Brady affirms that this manuscript is an honest, accurate, and transparent account of the study being reported; that no important aspects of the study have been omitted; and that any discrepancies from the study as planned (and, if relevant, registered) have been explained.

## Data Availability

The data that support the findings of this study are available on request from the corresponding author. The data are not publicly available due to privacy or ethical restrictions. Julianne E. Brady had full access to all of the data in this study and takes complete responsibility for the integrity of the data and the accuracy of the data analysis.
